# Employing an open-source tool to assess astrocyte tridimensional structure

**DOI:** 10.1007/s00429-016-1316-8

**Published:** 2016-09-30

**Authors:** Gabriela Tavares, Manuella Martins, Joana Sofia Correia, Vanessa Morais Sardinha, Sónia Guerra-Gomes, Sofia Pereira das Neves, Fernanda Marques, Nuno Sousa, João Filipe Oliveira

**Affiliations:** 1 Life and Health Sciences Research Institute (ICVS), School of Health Sciences, University of Minho, Campus de Gualtar, 4710-057 Braga, Portugal; 2 ICVS/3B’s, PT Government Associate Laboratory, Braga, Guimarães, Portugal; 3 DIGARC, Polytechnic Institute of Cávado and Ave, 4750-810 Barcelos, Portugal

**Keywords:** Astrocyte, Structure, Reconstruction, GFAP, Immunofluorescence

## Abstract

Astrocytes display important features that allow them to maintain a close dialog with neurons, ultimately impacting brain function. The complex morphological structure of astrocytes is crucial to the role of astrocytes in brain networks. Therefore, assessing morphologic features of astrocytes will help provide insights into their physiological relevance in healthy and pathological conditions. Currently available tools that allow the tridimensional reconstruction of astrocytes present a number of disadvantages, including the need for advanced computational skills and powerful hardware, and are either time-consuming or costly. In this study, we optimized and validated the FIJI-ImageJ, Simple Neurite Tracer (SNT) plugin, an open-source software that aids in the reconstruction of GFAP-stained structure of astrocytes. We describe (1) the loading of confocal microscopy Z-stacks, (2) the selection criteria, (3) the reconstruction process, and (4) the post-reconstruction analysis of morphological features (process length, number, thickness, and arbor complexity). SNT allows the quantification of astrocyte morphometric parameters in a simple, efficient, and semi-automated manner. While SNT is simple to learn, and does not require advanced computational skills, it provides reproducible results, in different brain regions or pathophysiological states.

## Introduction

Astrocytes are the most numerous cell type in the brain, and much has recently been discovered about their physiological relevance in the past few decades (Wang and Bordey 2008; Parpura et al. 2012; Araque et al. 2014; Volterra et al. 2014; Khakh and Sofroniew 2015; Oliveira et al. 2015). One of the most important discoveries regards the ability of astrocytes to maintain dialog with neurons, resulting in synaptic modulation. This dialog is possible due to morphologic features of the astrocytes, such as complex ramification, which allows close apposition of astrocytic processes to functional synapses and blood vessels. At these sites, astrocytes sense, process, and release neuroactive molecules to modulate synaptic communication and blood flow (Perea et al. 2009; Reichenbach et al. 2010; Araque et al. 2014), ultimately impacting network function and behavior processing (Oliveira et al. 2015). Astrocyte morphology is highly heterogenic (Matyash and Kettenmann 2010; Oberheim et al. 2012; Khakh and Sofroniew 2015). The idea that astrocytic process arbors vary consistently among species, brain regions, and physiological states is widely accepted (Bushong et al. 2002; Oberheim et al. 2009; Jinno 2011; Nash et al. 2011; Oberheim et al. 2012; Saur et al. 2014; Sofroniew 2015; Burda et al. 2016; Ghézali et al. 2016). Cell morphologies may be distinct in different astrocyte populations or physiological states in a healthy brain, correlating to specific molecular markers (Doyle et al. 2008; Jinno 2011). Moreover, astrocyte morphology is largely affected under pathological conditions. For instance, in some pathologies astrocytes undergo a dramatic increase in the process thickness and ramification named astrogliosis (Nash et al. 2011; Oberheim et al. 2012; Hol and Pekny 2015; Sofroniew 2015; Kulkarni et al. 2015; Yang and Wang 2015; Burda et al. 2016; Rodríguez-Arellano et al. 2016). Such structural alterations in astrocytes were shown to have functional consequences (Haber et al. 2006; Oberheim et al. 2012; Perez-Alvarez et al. 2014; Medvedev et al. 2014; Khakh and Sofroniew 2015; Heller and Rusakov 2015).

Assessing astrocyte structure and morphology requires complementary approaches to assess varying levels of detail (main process arbor versus fine process). Currently, there are multiple tools available to analyze and quantify the extremely complex astrocyte morphology (Parekh and Ascoli 2013; Kulkarni et al. 2015). The choice between options is normally based upon both the level of detail needed to answer the scientific question and the resources that are available. On one hand, a thorough, tridimensional analysis of main and fine processes may require advanced computational skills and powerful hardware (e.g., farsight-based tools). On the other hand, commercially available tools are rather user-friendly, yet they are costly and time-consuming [e.g., Neurolucida (MBF Bioscience, USA), Imaris (Bitplane, Switzerland)]. Taking these factors into account, we applied the open-source tool, Simple Neurite Tracer (SNT) (Longair et al. 2011), to reconstruct tridimensional arbors of astrocytic main processes. This method allowed to semi-automatically screen the astrocyte structure in a large number of samples, in a simple, effective and costless manner. The brain samples were stained against glial fibrillary acidic protein (GFAP), which is the intermediate filament protein that is used extensively as a specific marker for astrocyte main processes, whose expression is tightly related to morphological alterations (Wilhelmsson et al. 2006; Oberheim et al. 2012; Hol and Pekny 2015; Sofroniew 2015; Kulkarni et al. 2015; Yang and Wang 2015). Herein, we describe and validate the optimized procedure of (1) the loading of confocal microscopy Z-stacks, (2) the selection criteria, (3) the reconstruction process, and (4) the post-reconstruction analysis of morphological features (process length, number, thickness, and arbor complexity) in both mice and rat models.

## Materials and methods

### Simple Neurite Tracer

**Fig. 1:**
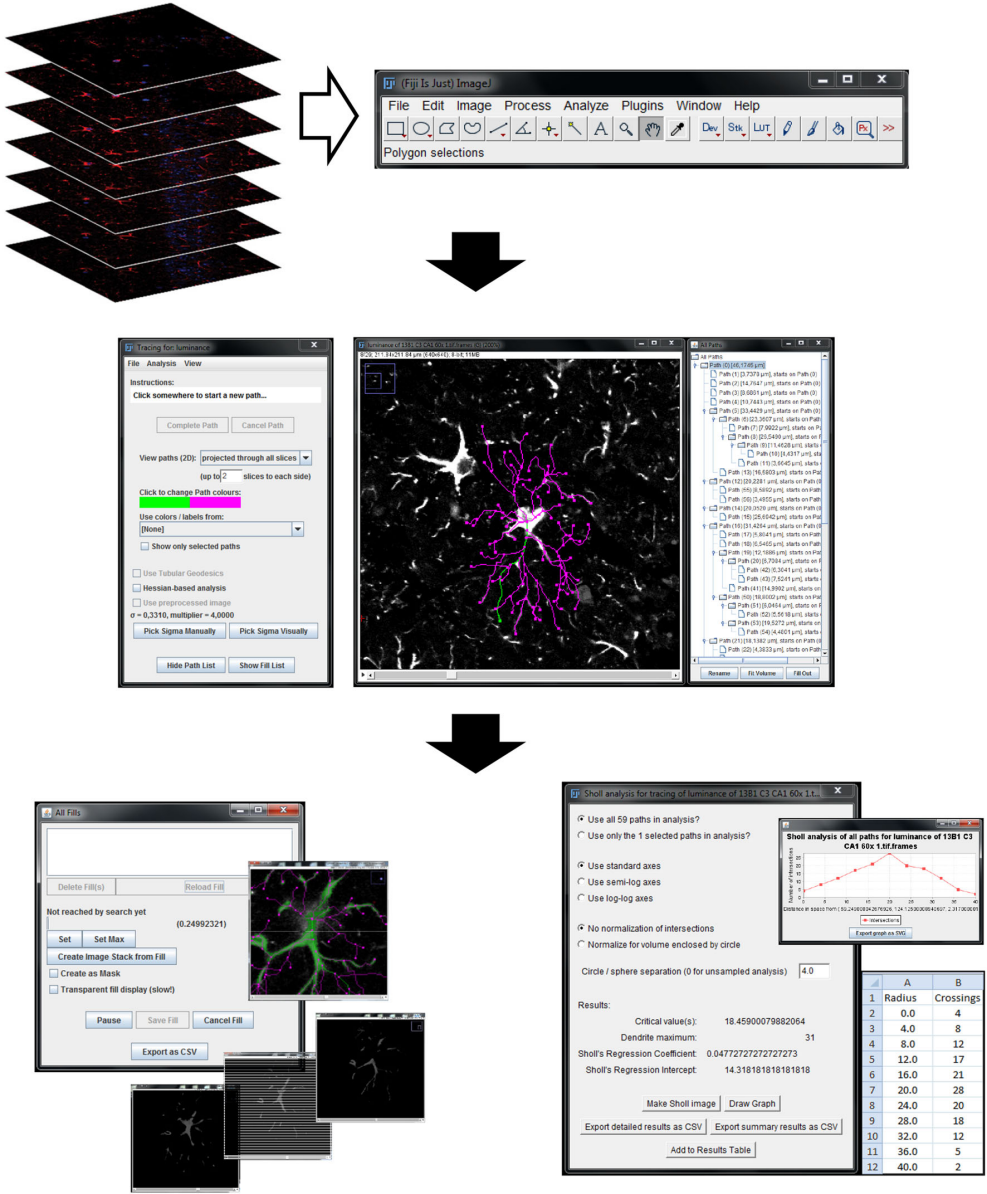
Reconstruction of astrocyte 3D morphology using SNT. Upload Z-stack of confocal images to Fiji software for astrocytic reconstruction (*upper panel*); Simple Neurite Tracer (SNT) menu and 3D astrocytic reconstruction example with all traced processes discriminated at “All paths window” (*middle panel*); thickness and Sholl analysis windows (*lower panel*)

Simple Neurite Tracer (SNT), a free software plugin distributed by Fiji-ImageJ, was developed by Mark Longair and his colleagues (2011) (Fig. 1). The free download and documentation are available at “http://fiji.sc/Simple_Neurite_Tracer”. SNT was originally designed to trace neurites, allowing the semi-automated tracing of tube-like structures in confocal microscope Z-stacks. To use SNT to reconstruct an astrocyte, the user must select points along the midline of the astrocytic process. If a pathway actually exists between the points, the software will connect them, even if they are distant from each other. This semi-automated process helps avoid reconstruction errors and simultaneously ensures correct process tortuosity.

**Fig. 2:**
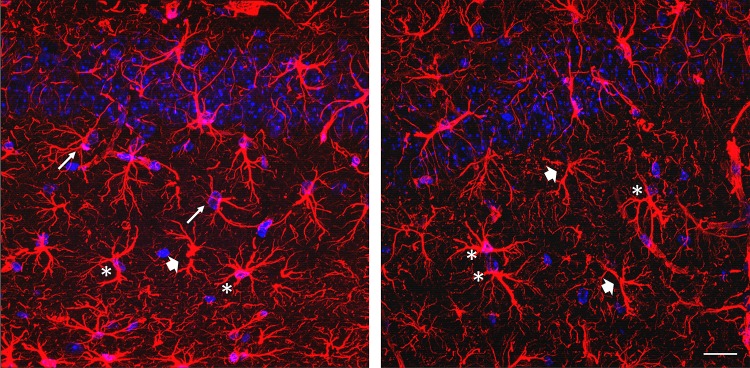
Astrocytes selection criteria for 3D morphology reconstruction using SNT. Representative GFAP–DAPI staining micrographs (max projection) showing GFAP^+^ cell suitable for 3D reconstruction (*asterisk*) and GFAP^+^ cells that fail the criteria for astrocytic reconstruction due to lack of nuclei (*thick arrows*) or the presence of two nuclei in the same GFAP^+^ structure (*thin arrows*); *scale bar* 50 µm

**Fig. 3:**
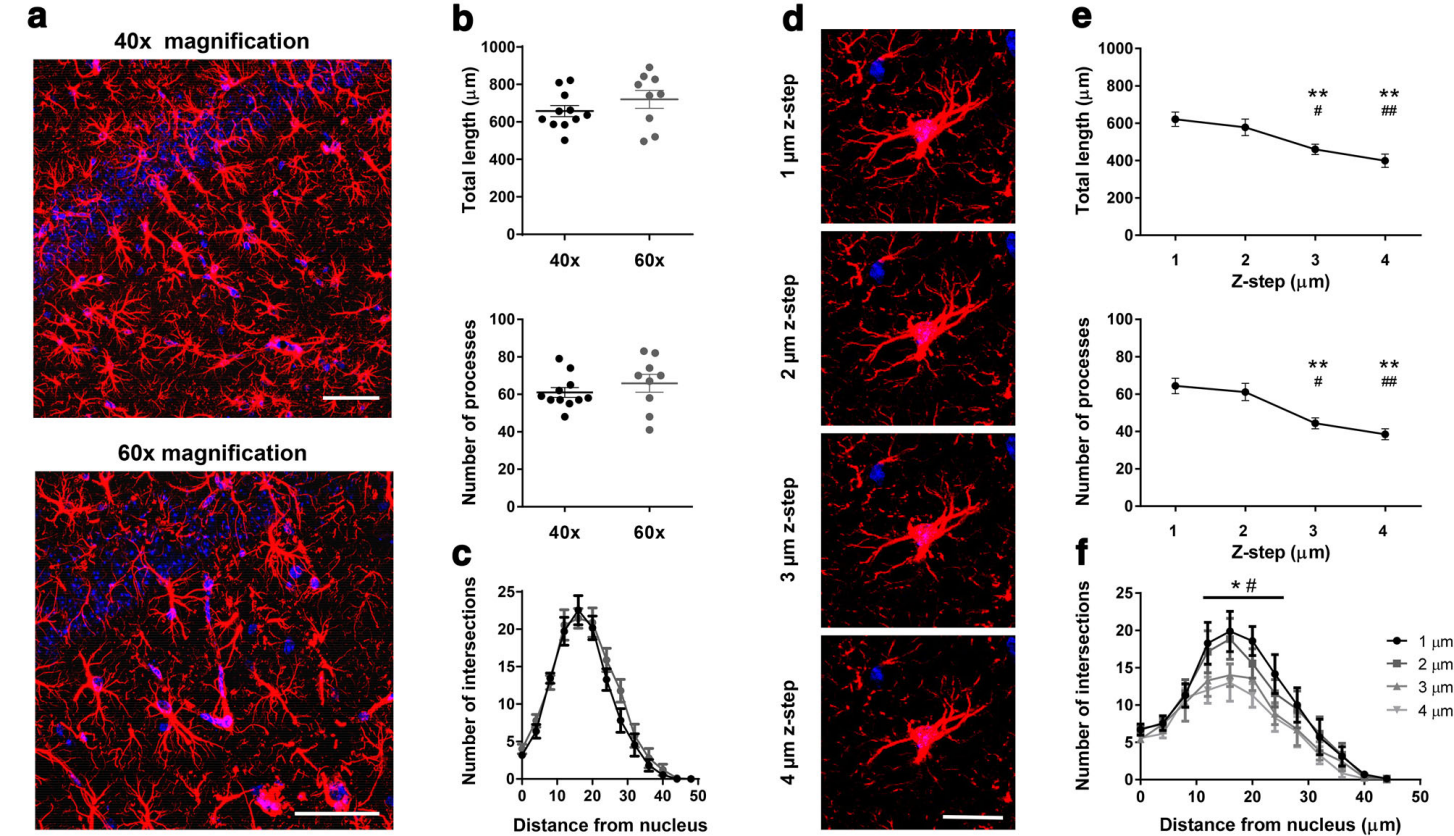
Suitable image Z-stack properties for 3D reconstruction of astrocytes. **a** Representative GFAP–DAPI staining micrograph (max projection) under 40× and 60× magnification; *scale bar* 50 µm. **b** Similar results of astrocytic morphology were obtained from reconstruction at 40× and 60× magnification, both for total length and number of processes. **c** At process complexity level, no differences were found between magnifications. **d** Representative micrograph of GFAP^+^ cell (max projection) showing decreased detail of astrocytic morphology information with increased Z-step interslice interval (1, 2, 3, 4 µm, at 60× magnification); *scale bar* 20 µm. **e** No statistically significant differences were found between 1 and 2 µm Z-step interval; 3 and 4 µm leads to a significant loss of detail in astrocytic morphology and unreliable measures of total length and number of processes. **f** Sholl analysis confirms loss of detailed information regarding process arbor complexity at higher Z-step interslice intervals. Data plotted as mean ± SEM. *p < 0.05; **p < 0.01 different from 1 µm; ^#^ *p* < 0.05 and ^##^ *p* < 0.01 different from 2 µm

**Fig. 4:**
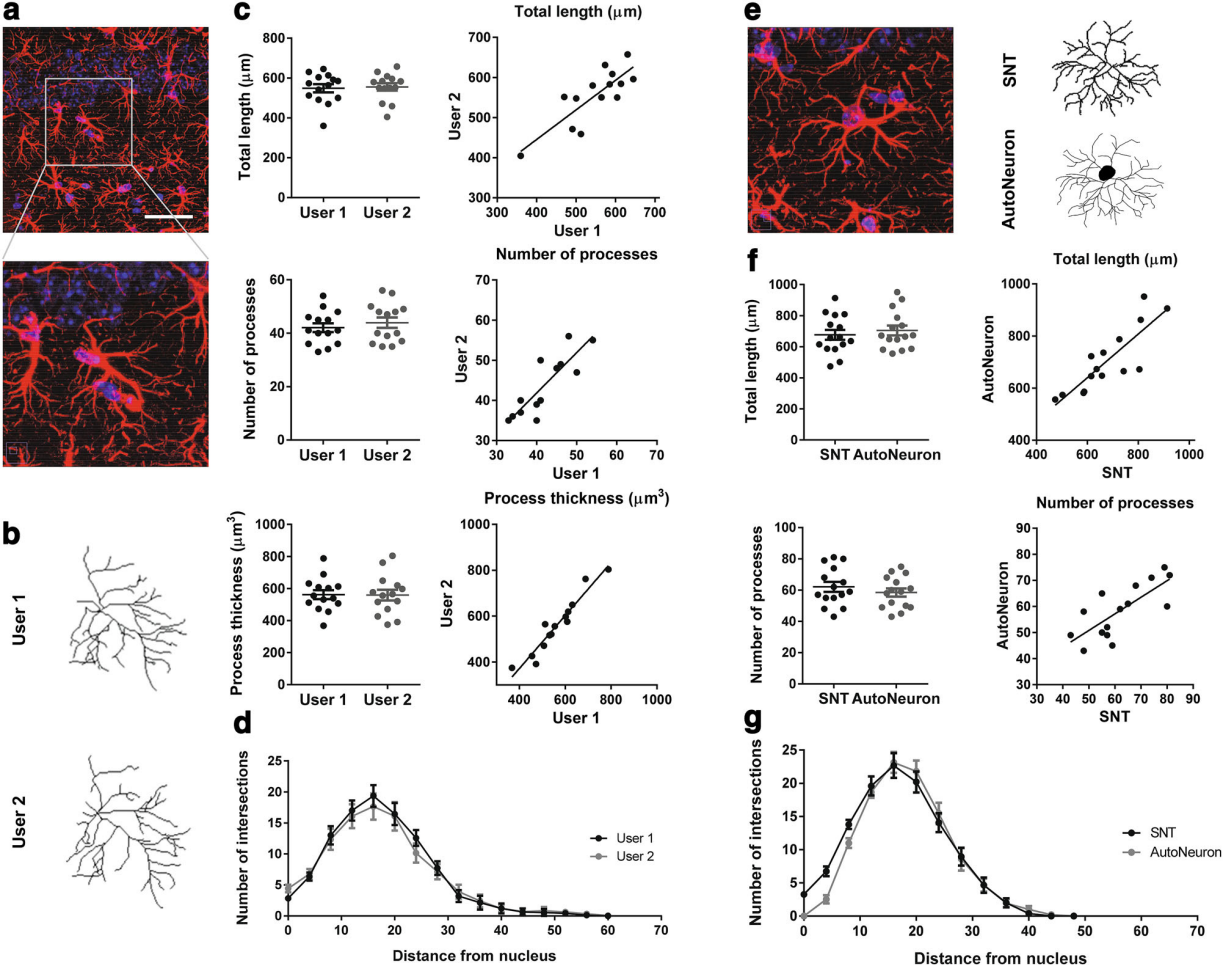
Simple Neurite Tracer is a reliable tool to reconstruct astrocyte process arbors. **a** Representative GFAP–DAPI staining micrograph (max projection) under 60× magnification, showing detailed astrocytic morphology; *scale bar* 50 µm. **b** Representative images of 3D astrocytic morphology reconstruction obtained from different users with SNT. **c** Similar morphometric results (astrocytic total length, number of processes and process volume) were obtained from different users’ 3D astrocytic reconstructions. **d** Sholl analysis shows similarity of process arbor complexity of reconstructed astrocytes from different SNT users. **e** Representative GFAP^+^ cell micrograph (max projection) and its respective reconstruction obtained from AutoNeuron and SNT; *scale bar* 50 µm. **f** Astrocytic reconstructions obtained from AutoNeuron and SNT confirm similarity between total length and number of processes. **g** Sholl analysis confirms similarity between reconstructed astrocytic morphologies at a detailed level of arbor complexity

**Fig. 5:**
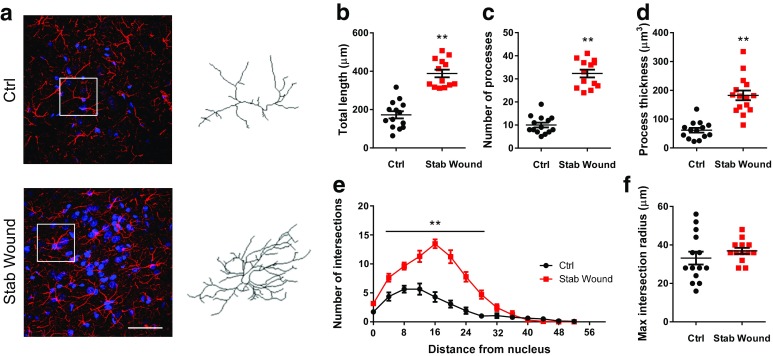
Simple Neurite Tracer discriminates altered morphology states. **a** Representative micrographs of GFAP–DAPI staining (max projection) of rat brain sections, including the medial prefrontal cortex from control (Ctrl) and stab wound injury (Stab Wound) animals with a representative reconstructed astrocyte from SNT for each condition, respectively; *scale bar* 50 µm; **b** stab animals present astrocytes with greater total length, **c** increased number of processes, and **d** GFAP process volume. **e** Sholl analysis results demonstrate increased arbor complexity in stab animals. **f** No differences were observed regarding maximum extension of astrocytic processes between groups. Data plotted as mean ± SEM. *p < 0.05 and **p < 0.01

We describe here how to use SNT intuitive workflow to perform the morphometric analysis of astrocytic processes by quantifying their total length, number, thickness, and intersections at concentric spheres originating from the soma (Sholl analysis). We examined confocal Z-stack images, obtained from rodent brain tissue stained by immunofluorescence for GFAP (astrocyte main processes) and DAPI (cell nuclei). We chose to use confocal Z-stacks because fluorophores suffer extensive photobleaching during long-lasting manual reconstruction of their arbor processes. Details on the animal models used and tissue preparation are given below. After the immunostaining, Z-stacks of confocal images (tif format), including two channels (red, GFAP; blue, DAPI), were obtained via Olympus FV1000 laser scanning microscope. A resolution of 640 × 640 px was achieved using a 60× objective (PlanApo N, N.A. 1.42; oil; field size 211.51 × 211.51 µm; 0.33 µm/px; Figs. 2, 3, 4, 5) and 1024 × 1024 px using a 40× objective (UPlanSApo, N.A. 0.90; dry; field size 317.13 × 317.13 µm; 0.31 µm/px; Fig. 3a), to work with a similar image resolution. The acquisition settings were the following: scanning speed, 4 µs/px; pinhole aperture, 110 µm; GFAP, excitation = 559 nm, emission = 618 nm; DAPI, excitation = 405 nm, emission = 461 nm; pinhole aperture = 110 µm. The images analyzed were not post-processed.

### Astrocyte selection criteria for reconstruction

Astrocytes were readily identified by their characteristic GFAP-positive bushy shape, displaying thicker processes around the DAPI-stained nucleus. The selection criteria used (examples in Fig. 2) are listed below:

1. GFAP-stained structure encloses a single DAPI-stained nucleus.
2. The main structure does not have truncated processes.
3. Reconstruct the first ten astrocytes per animal that fulfill the above-mentioned criteria (maximum of four astrocytes per randomly selected Z-stack).

### Step-by-step reconstruction of an astrocyte from a previously acquired Z-stack of images

1. Import the image stack into Fiji (Menu: File\Import\Image Sequence); select one image from the stack; press “Open”; in the “Sequence options” verify that the information provided is correct, otherwise update.
2. Open SNT (Menu: Plugins\Segmentation\SNT); press “Yes” to convert every RGB image to 8-bit luminance; choose 3D visualization options; one may deselect all options to work in a simpler desktop (Fig. 1, middle panel).
3. Follow the reconstruction instructions available at “http://fiji.sc/Simple_Neurite_Tracer:_Step-By-Step_Instructions” to trace the astrocyte selected. Keyboard shortcuts were found very useful. After the selection of a distant point within a process, SNT will automatically suggest the process midline, respecting the process tortuosity. Due to the heterogeneous structure of the astrocytic soma, and its relationship to the main processes, the center of the DAPI staining was always defined as the starting point. Every main process must be reconstructed from this starting point, to perform the Sholl analysis.
4. The information of the reconstructed astrocytes (list of paths and individual path lengths) appears in the “All paths” window. The paths located in the first tree level represent the main processes (Fig. 1, middle panel).

### Saving and exporting

- The “All paths” window data may be saved as CSV file (SNT Menu: File\Export as CSV).
- The 3D reconstructed traces may be saved as an SWC file (SNT Menu: File\Save traces file). This file may be opened and edited anytime, yet the original Z-stack should be pre-loaded in Fiji, repeating steps 1 and 2.
- The 3D reconstructed traces may be exported to a 2D image format:

1. After reconstruction step 3, go to SNT Menu: Analysis\Make line stack.
2. Make Z-projection at Fiji Menu: Image\Stacks\Z project (Max intensity).
3. Invert black/white at Fiji Menu: Edit\Invert.
4. Save as image at Fiji Menu: File\Save as.

### Volume analysis

1. Select all paths and press “Fill out” (detailed instructions under “http://fiji.sc/Simple_Neurite_Tracer:_Basic_Instructions#Filling_Out_Neurons”).
2. In the “All fills” window (Fig. 1, lower panel, left), set the threshold to a value that produces an adequate fill of the GFAP staining across a considerable number of cells, respecting both thicker and thinner processes (0.05 yielded reproducible results across astrocytes of four different sets of rats and mice in our experiments); press “Set”.
3. Export the volume data to a CSV file.

### Sholl analysis

1. Select the first traced path in the “All paths” window and press Ctrl + Shift to follow the path. Select the starting point located in the center of the DAPI staining. Press Ctrl + Shift + A to open the “Sholl analysis” window (detailed instructions under “http://fiji.sc/Simple_Neurite_Tracer:_Sholl_analysis”).
2. In the “Sholl analysis” window (Fig. 1, lower panel, right), select “Use all paths” and “sphere radius”. A radius of 4 µm was sufficient to provide enough morphologic detail for GFAP-stained astrocytes in our study (Figs. 3, 4, 5).
3. Export the data as a CSV file.

### Data interpretation

1. Total process length: sum of the length of all individual paths, obtained from the “All paths” window.
2. Number of processes/endings: number of individual paths, obtained from the “All paths” window.
3. Process thickness: estimated from the GFAP thickness; provided by the “Fill Out” analysis.
4. Morphology complexity: estimated from the number of intersections at each radial distance from the starting point (DAPI-stained nucleus); provided by the Sholl analysis.

### Animals, surgical procedures and tissue preparation

All experiments were performed in accordance with the European Directive 2010/63/EU, of 22 September and DGAV Decreto-Lei No. 113/2013, of 7 of August. All animals were group housed in standard cages under defined laboratory conditions (light/dark cycle 8 a.m. to 8 p.m.; room temperature 22 °C; ad libitum access to food and water). Mice and rat models were used to confirm the suitability of SNT for astrocyte reconstruction in both species. To validate the method, hippocampal slices obtained from C57BL/6J wild-type mice (*n* = 4) (Charles River, Barcelona, Spain) were stained (Figs. 3, 4). The stab wound injury model was performed on Wistar Han rats (*n* = 2 per group) to induce astrogliosis (Sofroniew 2015) and confirm the ability of SNT to discriminate morphological changes in astrocytes under this condition (Fig. 5).

The stab wound injury was performed in a similar manner to that previously defined (Lima et al. 2014). Briefly, rats were deeply anesthetized with a mixture of ketamine (75 mg/kg, i.p.; Imalgene 1000, Merial, EUA) and medetomidine (0.5 mg/kg, i.p.; Dorbene Vet, Pfizer, EUA). A 30 G needle was stereotaxically inserted into the medial prefrontal cortex, bilaterally, following the coordinates 3.0 mm posterior to bregma, ±0.6 mm lateral to the midline and 2.5 mm ventral to the skull surface, based on the Paxinos and Watson rat brain atlas (2005). Age- and sex-matched rats, not submitted to needle insertion, were considered as controls. At the end of the surgical procedure, the anesthesia was reversed with atipamezole (2 mg/kg i.p.; Antisedan, Pfizer) and the animals were given 6 days to recover. After the rest period, rats and wild-type mice were deeply anesthetized (for mice, the concentration of medetomidine was corrected to 1 mg/kg in the mixture) and immediately intracardially perfused with saline, followed by 4 % paraformaldehyde solution [(PFA, 0.1 M, pH 7.4, in phosphate saline buffer (PBS)]. Brains were removed and immersed in 4 % PFA (48 h), followed by 1 week in a 30 % sucrose PBS buffer (at 4 °C). Brains were then frozen by immersion in isopentane (BDH Prolabo; cooled in liquid nitrogen) in Neg-50 frozen section medium (Thermo Scientific, EUA) and stored at −20 °C until sectioning.

The immunohistochemistry protocol was performed in cryosections (20 µm-thick) of mice (stratum radiatum, CA1, dorsal hippocampus; Figs. 2, 3, 4) and rat (layers 3–5, medial prefrontal cortex; Fig. 5) brains. Tissue slices were hydrated with PBS for 10 min and then permeabilized with PBS-T 0.3 % (0.3 % triton X-100, Sigma Aldrich, USA, in PBS) for 10 min. Antigen retrieval was then performed by immersing the slices in pre-heated citrate buffer (10 mM, pH 6.0; Sigma Aldrich, USA) during 20 min at microwave low potency. Once cooled, slices were rinsed in PBS and then in 10 % fetal bovine serum (FBS) in PBS. The slices were then incubated at room temperature for 30 min. The slices were incubated in the primary antibody, rabbit polyclonal anti-GFAP (1:200; Dako, Denmark) diluted in PBS-T 0.3 % 4 % FBS, and then incubated at 4 °C overnight. Next morning, tissue slices were rinsed in PBS and incubated with a secondary antibody, Alexa Fluor^®^ 594 goat anti-rabbit (1:1000; Molecular Probes^®^, Invitrogen, USA) diluted in PBS during 90 min at room temperature, protected from light. DAPI staining was performed by a 10-min incubation (1:1000; Invitrogen, USA), followed by several rinses with PBS. Coverslips were mounted using Immu-Mount™ (Thermo Scientific, USA).

### Statistical analysis

Results are presented as mean ± SEM (standard error of the mean). The statistical significance was observed for a confidence level of 95 %. All data sets passed the Shapiro–Wilk normality test for Gaussian distributions. Accordingly, parametric tests were applied throughout. *t* tests were used to compare astrocytic total length, number of processes and process thickness between groups (unpaired, Figs. 3, 5; paired, Fig. 4). One-way analysis of variance (ANOVA) was applied to compare astrocytic morphologies (total length and number of processes) obtained by decreasing the Z-step intervals (Bonferroni post hoc comparisons; Fig. 3e). Two-way ANOVA was used to compare Sholl analysis data between astrocytic reconstructions (between experimental groups and along the radial distances; Sidak post hoc comparisons; Figs. 3, 4, 5). Pearson correlation coefficients were calculated to compare distributions of data obtained through two different reconstruction programs. Additionally, the degree of consistency between two independent users was evaluated by calculating Pearson coefficients (Fig. 4). Statistical analysis was performed by using GraphPad Prism 6 (GraphPad Software Inc., USA).

## Results

To validate the reconstruction of GFAP-stained astrocytic processes using SNT, we: (1) defined the properties of a correct image Z-stack; (2) compared the SNT reconstruction using multiple users and programs; (3) used a model of astrogliosis to assess the astrocytes of differing complexity.

### Suitable image Z-stack properties for 3D reconstruction of astrocytes

To define the minimal image Z-stack requirements so as to ensure a reproducible reconstruction, two different sets of images were analyzed by an experienced researcher: Z-stacks acquired using two different magnification objectives (40× at 1024 × 1024 px, 0.31 µm/px; 60× at 640 × 640 px, 0.33 µm/px for comparable structural detail); Z-stacks acquired at different interslice intervals (Fig. 3). The reconstruction of astrocyte structure in Z-stacks of 40× and 60× yielded similar results for total process length (*t*_18_ = 1.17; *p* = 0.26), number of processes (*t*_18_ = 0.94; *p* = 0.36), and process arbor complexity (*F*_1,18_ = 1.86; *p* = 0.19; Fig. 3a–c; 3–4 Z-stacks for each magnification). It is noteworthy that although the results are not significantly different, the level of detail of the images acquired with 60× magnification allowed a more confident reconstruction, namely by users with low experience.

Z-stacks were obtained at four different interslice intervals: 1, 2, 3 and 4 µm (Fig. 3d–f; four Z-stacks each). Analysis of total length and number of processes demonstrated that while Z-intervals of 2 µm still provide enough Z resolution for GFAP-staining reconstruction, 3 and 4 µm intervals significantly reduced the reliability of the results (at least *p* < 0.05). These results were supported by the Sholl analysis, which pointed out a crucial reduction of arbor complexity between 12 and 28 µm from the astrocyte soma (at least *p* < 0.05 between 1 or 2 µm, and 3 or 4 µm). The number of intersections at radius zero represents the number of main processes that leave the DAPI-stained nuclei; the lack of differences between Z-stacks at this radius is expected since thicker processes are still present at lower Z-resolutions.

Based on these results, most of the subsequent work was performed using a magnification of 60× (640 × 640 px) and a Z-step of 1 µm to allow a safety margin for morphometric reliability.

### Simple Neurite Tracer is a reliable tool to reconstruct astrocyte process arbors

To assess the reliability and reproducibility of the data, two comparative analyses were performed: astrocytes from a set of image Z-stacks were reconstructed by two independent users with different degrees of experience; one experienced researcher reconstructed the same set of Z-stacks using two different tools: SNT and Neurolucida (MBF Bioscience).

Although reconstruction presents an expected degree of subjective variability, the data obtained by the two users confirm the lack of statistically significant differences for all parameters analyzed: total process length (*t*_13_ = 0.56; *p* = 0.59), number of processes (*t*_13_ = 1.84; *p* = 0.09), and process thickness (*t*_13_ = 0.30; *p* = 0.77; six Z-stacks; Fig. 4a–c). Moreover, these data sets indicated a high degree of interrater consistency for the three parameters analyzed: total length (*r* ^2^ = 0.67 and *p* < 0.01), number of processes, (*r* ^2^ = 0.73, *p* < 0.01) and process thickness (*r* ^2^ = 0.92, *p* < 0.01). Accordingly, the Sholl analysis pointed out an indistinguishable morphological complexity between the two users (*F*_1,26_ = 0.10, *p* = 0.75; Fig. 4d).

In the next step, SNT efficiency was tested by performing 3D reconstructions of a same set of brain slices using an extension module of Neurolucida, AutoNeuron, and three Z-stacks of confocal images, in parallel with SNT. This software is widely used and recognized for neuronal reconstruction. Due to the complex and non-linear distribution of structures in the GFAP staining, we employed the manual reconstruction procedure. Once more, SNT proved statistically identical to the commercially available software for total process length (*t*_14_ = 1.57, *p* = 0.14), number of processes (*t*_14_ = 1.73, *p* = 0.11), and Sholl analysis (*F*_1,28_ = 0.86, *p* = 0.37; Fig. 4e–g). The variation observed in the Sholl analysis at lower radial distances is explained by the different representation of the cell soma by AutoNeuron, as depicted in Fig. 4e. Moreover, the measures of total process length and number of processes displayed a high degree of correlation (*r* ^2^ = 0.71, *p* < 0.01 and *r* ^2^ = 0.57, *p* < 0.01, respectively; Fig. 4f, right panel).

### Simple Neurite Tracer discriminates altered morphology states

Finally, after verifying the reliability of SNT to reconstruct 3D astrocytic structures, the authors intended to verify the sensibility of SNT in discriminating between varied levels of arbor complexity. Confocal Z-stacks were obtained from brain slices of a stab wound rat model. This model presents extensive astrogliosis surrounding the stab wound site, a phenomenon classically characterized by an increase in the thickness and complexity of the astrocytic process arbors (Wilhelmsson et al. 2006; Sofroniew 2015) (Fig. 5a). In this evaluation, the structure of astrocytes reconstructed from brain tissue of the stab wound region was compared to that obtained from the reconstruction of astrocytes from control animals without lesions.

As indicated by the data, the reconstruction of astrocytes using SNT allowed a clear dissection of morphological alterations. First, astrocytes from the lesion site display a drastic increase in the total process length (about twofold; *t*_25_ = 7.78; *p* < 0.01), number of processes (about threefold; *t*_25_ = 11.60; *p* < 0.01), and process thickness (about threefold; *t*_25_ = 6.91; *p* < 0.01; Fig. 5b–d). Besides the increased process arbor complexity, indicated by the large number of intersections in stab wound astrocytes at the same radius (about threefold; *F*_1,25_ = 68.35; *p* < 0.01 where indicated; Fig. 5e), the Sholl analysis pointed out a shift in the intersection curve to the right, which indicates enhanced complexity at sites more distant from the soma of the astrocytes in the stab wound model. Interestingly, despite the increased process arbor complexity, GFAP-stained processes of astrocytes, at lesion sites, extend to distances similar to those of controls (Fig. 5f; *t*_25_ = 0.99; *p* = 0.33).

In summary, SNT discriminates between different morphologic measures, allowing a better characterization of the astrocytes under pathological conditions.

## Discussion

The reconstruction of GFAP-stained structures by SNT allows the simple, efficient, and semi-automated quantification of astrocyte morphometric parameters, such as total process length, number of processes, process thickness, and arbor complexity. SNT offers several technical advantages, such as: interface simplicity, intuitive workflow, avoidance of image pre-processing or post-analysis, availability for Windows, Mac OS and Linux, simple interaction with complementary Fiji plugins, importation of numerous image formats, exportation of data in different file formats, and, most importantly, it is available for free. It is important to note, however, that this method excludes astrocytic morphological details that occur on a nanoscale level (Bushong et al. 2002; Rusakov et al. 2014). To access this level of detail, one must employ a much more powerful reconstruction tool, coupled with a higher level of image resolution. Nevertheless, this method presents a powerful tool for main structure reconstruction at the microscale level, which is useful, for instance, when studying pathological states characterized by astrogliosis. A second natural limitation of this method stems from the slice thickness used. In this work, we analyzed 20 µm-thick brain slices due to the variable levels of antibody penetration observed in thicker brain slices, which would result in a heterogeneous staining of deeper astrocytic structures. The user should bear in mind the expected process truncation at the slice limits, which might bias the results in cases of morphological anisotropy. Therefore, we strongly recommend a comprehensive sampling of the cells using appropriate controls, so that every cell population is duly characterized, despite its polarity or complexity of the process arbor.

During the first stage, we analyzed a number of image Z-stacks to identify the minimum requirements for astrocytic reconstruction. The data obtained reveals that using the 40× objective lens and/or using 2 µm interslice intervals will provide acceptable results. Both configurations increase the speed of the image acquisition process and may be of relevance when dealing with a large number of samples. Nevertheless, we believe that the use of the 60× objective (even with a lower resolution) and 1 µm interslice interval provides a safer margin for morphometric reliability as well as a higher level of tracing confidence.

During the second stage, we assessed the reliability of the SNT reconstructions. By comparing the reconstructions of different users and different tools, we confirmed a high degree of data reproducibility in both cases, for the measures analyzed. It is noteworthy that, due to the semi-automated nature of SNT, incorrect process reconstructions were avoided. Semi-automatic reconstruction methods provided a low error while maintaining the integrity of the reconstruction process among users, saving time (even in comparison with fully automated methods, which require post-tracing verification of the end result) (Myatt et al. 2012; Kulkarni et al. 2015).

Finally, we performed reconstructions of astrocytes in brain tissue of a rat model of astrogliosis. The reconstruction analysis provided detailed information on the variation of each parameter analyzed. The number of processes and arbor thickness increased more greatly than did the total length, indicating that, in this model, the ramification, and not the elongation, is more greatly affected. This data supports previous observations (Wilhelmsson et al. 2006; Sofroniew 2015). Furthermore, SNT allowed to discriminate between differential levels of detail, which makes SNT a valuable tool in the study of astrocyte structure in a large number of pathological states.

In the last several decades, a series of developments in microscopy and molecular biology techniques have revealed a huge level of morphological detail for brain cells, namely, astrocytes (Pool et al. 2008; Donohue and Ascoli 2011; Parekh and Ascoli 2013; Kulkarni et al. 2015). The need for quantification of morphometric properties has triggered the development of a range of informatics tools. However, the available methods for astrocyte morphology reconstruction are either sophisticated—not useful to life science researchers with limited programming experience—or expensive (Pool et al. 2008; Parekh and Ascoli 2013; Kulkarni et al. 2015). To choose the most suitable tool, a researcher should balance quality and resolution of the samples, available time, and cost. Thus, assessing astrocyte morphology requires knowledge of the level of resolution necessary for addressing the scientific question. Herein, we have demonstrated that the use of SNT will add value to the field, since it may be easily employed by any researcher to characterize the main astrocyte structure under varied physiological and pathological conditions, providing reproducible results using minimal computational skills or hardware capacity.
